# Trismus Nasaentium

**Published:** 1863-02

**Authors:** 


*Trismus Nascentium.*—GREENVILLE DOWELL, M. D., of Texas, in the Am. Journal, concludes from considerable experience and observation that *Trismus Nascentium* is produced:

1st. Most frequently from improper management of the cord, and congestion of blood in the umbilical vein; from this vein congestion spreads to the liver, and it becomes swollen and filled with dark blood, and the secretion of bile is entirely arrested.

2d. The next most frequent cause is the displacement of the occipital bone in parturition. We may always look for this cause in protracted labors.

3d. The retention of the meconium, and want of properly cleansing the skin when the child is first dressed.

4th. And lastly, it may be produced by any cause that will produce tetanus in old persons. Cold, exposure to drafts of cold air; foul odors, &c.

It generally comes on from the fifth to the twelfth day, most frequently the ninth. After three weeks he considers the child safe. In treatment he refers to the cause. If from displacement of the cranial bones, as suggested first by Dr. Sims, he removes it by position; if the occiput is at fault, on the side; if the parietal, on the back. Does not allow the position to be changed even during nursing.

Originating from the umbilicus, he rubs the abdomen with an ointment of Hydrarg. Protiodid gr. x to ℥j of Lard, giving internally from one to five grs. of Calomel. Opiates, Assafœtida, &c., appear to do no good. The warm mustard bath nearly always temporarily relieves the spasms.

The third and fourth causes indicate with sufficient clearness the proper remedies.

Proper management of the cord at birth he thinks of the very highest importance in the disease.

With regard to the second cause referred to by Dr. D., or Sims' disease as it might be called, we refer our readers to an article by Dr. Brainard, in this JOURNAL, vol. V. (1862) p. 4." "Tendency to a Vacuum in Cerebro-Spinal Diseases." The cause is seen to be a feeble action of the heart, or a peculiar modification of the respiratory movements, or both. A strong inspiration tends to exhaust the cerebro-spinal cavity, and hence the overlapping, when the laryngeal aperture is partially or wholly closed. Of course some cases are produced during a severe parturition by the great pressure applied to the head of the child naturally, or it may be by use of extracting instruments.

But why do these cases occur more frequently South than North?

